# Willingness to Act upon Beliefs about ‘Treatment as Prevention’ among Australian Gay and Bisexual Men

**DOI:** 10.1371/journal.pone.0145847

**Published:** 2016-01-07

**Authors:** Benjamin R. Bavinton, Martin Holt, Andrew E. Grulich, Graham Brown, Iryna B. Zablotska, Garrett P. Prestage

**Affiliations:** 1 Kirby Institute, University of NSW Australia, Sydney, Australia; 2 Centre for Social Research in Health, University of NSW Australia, Sydney, Australia; 3 Australian Research Centre in Sex, Health and Society, La Trobe University, Melbourne, Australia; David Geffen School of Medicine at UCLA, UNITED STATES

## Abstract

HIV ‘treatment as prevention’ (TasP) is highly effective in reducing HIV transmission in serodiscordant couples. There has been little examination of gay and bisexual men’s attitudes towards TasP, particularly regarding men’s willingness to act on beliefs about TasP. We conducted an online cross-sectional survey of Australian men in late 2012 to investigate knowledge and beliefs about new developments in HIV prevention. Amongst 839 men (mean age 39.5 years), men tended to disagree that TasP was sufficiently effective to justify reduced condom use, although HIV-positive men had more favourable attitudes. Only a minority of men were aware of any evidence for TasP; and one-quarter incorrectly believed that evidence for the effectiveness of TasP already existed for the homosexual population. One-fifth (20.5%) of men reported that they would be willing to have condomless anal intercourse with an opposite-status sexual partner when the HIV-positive partner was taking HIV treatments. Factors independently associated with such willingness were: HIV-positive serostatus, reporting any serodiscordant or serononconcordant condomless anal intercourse with a regular male partner in the previous six months, reporting any condomless anal intercourse with a casual male partner in the previous six months, and having greater beliefs in the effectiveness of TasP. This indicated that the men most willing to rely on TasP to prevent transmission were already engaging in higher risk practices. Biomedical HIV prevention represents a rapidly changing environment with new research as well as community and policy responses emerging at a fast pace. For men with serodiscordant sexual partners to successfully apply TasP to reducing transmission risk, more support and education is needed to enable better utilisation of TasP in specific relational and sexual contexts.

## Introduction

‘Treatment as prevention’ (TasP), the strategy of treating HIV-positive people with antiretroviral treatments (ART) to prevent onward sexual transmission of HIV [1–3], has been proven effective for heterosexual serodiscordant couples, where the transmission risk was reduced by 96% if the HIV-positive partner was on treatment [4]. Conversely, data on TasP within homosexual male serodiscordant couples, and for anal sex more generally, is promising, but more limited [5–7].

An international systematic review conducted in 2014 found only two articles concerning gay men’s attitudes about TasP [8–10]. In Australia, one national study has reported on two rounds of surveys conducted in 2011 and in 2013, which found that most men had sceptical views of the preventative benefits of HIV treatment [10, 11]. The majority, nonetheless, agreed that HIV-positive men should start treatment early, though mainly for their own health benefits. The authors noted that this potentially represented a mismatch between community attitudes and policy, given that many governments are proceeding apace with TasP-related strategies [11]. Similarly, one recent study of gay men in Vancouver found that less than ten percent of the men used undetectable viral load as a prevention strategy [12].

Previous attitudinal research has explored men’s understandings of the effectiveness of TasP in principle, that is, whether they believe HIV treatment prevents transmission. This research, however, has not addressed how likely men are to *act* on those beliefs. This analysis adds to the existing data by specifically extending the question of men’s understandings of TasP to address their willingness to incorporate it into their own sexual behaviour. It examines Australian gay and bisexual men’s (GBM) attitudes regarding TasP, utilising the concept of willingness to engage in serodiscordant condomless anal intercourse (CLAI) when the HIV-positive partner in that scenario is taking ART.

## Methods

### Procedures

We conducted a cross-sectional study of Australian GBM in late 2012, using an anonymous online survey platform (SurveyGizmo 3.0) to investigate knowledge and beliefs about recent developments in HIV prevention and testing, including TasP and pre-exposure prophylaxis (PrEP). Given that a major purpose of the survey was to test men’s knowledge about these new prevention tools, the survey provided limited information about them to participants. The data were collected before TasP was broadly discussed within Australian gay communities, and before any TasP studies had reported efficacy results specific to homosexually active men. The study was promoted through gay community events and venues, and online via advertisements on gay community websites, gay dating websites, and through gay community online media. No compensation for participation was provided. Participants were provided with information about the study at the start of the survey. As an anonymous online survey, consent forms were not completed as the act of voluntarily completing the survey was taken as consent, as per the guidelines of the Australian National Health and Medical Research Council. There were no special consenting procedures for participants aged less than 18 years; parental consent was not obtained for these participants. Ethics approval for the study and for this approach to consenting, including the inclusion of participants aged 16 and 17 years without parental consent, was provided by the Human Research Ethics Committees of the University of New South Wales.

### Participants

Men who lived in Australia, aged 16 or above, were eligible for participation if they identified as gay or bisexual, or reported having sex with another man in the previous year.

### Measures

The questionnaire included self-report items on: demographics (including year of birth, ethnicity, employment and education), sexuality, social engagement with gay men [13], sexual behaviour, condom use, relationships, HIV testing history, and serostatus. CLAI was assessed for both regular and casual male partners, for insertive and receptive sexual positions and for sex with withdrawal before ejaculation and ejaculation inside the rectum. Participants were asked about the HIV status of regular but not casual partners. These items were combined to create measures of: any CLAI with regular partners (CLAI-R); any CLAI with regular serodiscordant or serononconcordant partners; and any CLAI with casual partners (CLAI-C) in the previous six months.

The primary outcome variable in this analysis was the respondent’s willingness to engage in CLAI with an opposite-serostatus sexual partner if the HIV-positive partner in that scenario was taking ART. HIV-positive and non-HIV-positive men received different versions of the questions. Two items on willingness to engage in CLAI were asked: “How much do you agree or disagree that you would be willing to fuck (top) an HIV-positive man without a condom if he was taking anti-HIV medications?” and “How much do you agree or disagree that you would be willing to get fucked (bottom) by an HIV-positive man without a condom if he was taking anti-HIV medications?” (non-HIV-positive respondents’ versions). Each item was scored on a four-point Likert scale ranging from 1 = ‘strongly disagree’ to 4 = ‘strongly agree’; they were then dichotomised in ‘willing’ and ‘not willing’. The HIV-positive and non-HIV-positive versions of these items were combined to form variables on the respondent’s willingness to engage in serodiscordant CLAI when the HIV-positive partner was on ART in three scenarios: overall; when the HIV-negative partner was insertive; and when the HIV-negative partner was receptive.

Participants were asked about their knowledge of the existing TasP evidence; beliefs about the effectiveness of TasP; and belief about whether HIV-positive men should commence ART to reduce their chances of passing on HIV to others. Additionally, several sets of attitudinal questions based on previous research [14, 15] were included, utilising a four-point Likert scale ranging from 1 = ‘strongly disagree’ to 4 = ‘strongly agree’. These were used to formulate two reliable scales: (1) *Belief in Effectiveness of TasP Scale* (Cronbach’s α = 0.83); and (2) *Belief in Health Benefits of Treatments Scale* (Cronbach’s α = 0.70). All scale items were scored from 1 to 4 where higher scores indicated more favourable attitudes. Some items were worded differently for HIV-positive or HIV-negative/unknown men. Scale items are presented in Table 1.

**Table 1:** Individual scale items for total sample, and comparison between HIV-positive and non-HIV-positive men (bivariate).

	All men (n = 839)		Non-HIV-positive men (n = 674)		HIV-positive men (n = 165)		χ^2^	p-value
	Disagree (%)	Agree (%)	Disagree (%)	Agree (%)	Disagree (%)	Agree (%)		
***Belief in Effectiveness of TasP Scale***								
HIV-positive men who are on treatments are unlikely to pass on HIV if they fuck without a condom.	83.7	16.3	87.7	12.3	67.5	32.5	39.33	<0.001
An undetectable viral load makes it unlikely to pass on HIV.	66.9	33.1	72.1	27.9	45.7	54.3	40.91	<0.001
I fuck without condoms more often because of HIV treatments.	85.5	14.5	91.2	8.9	62.4	37.7	87.38	<0.001
HIV treatments take the worry out of sex.	90.0	10.0	94.5	5.5	71.4	28.6	76.55	<0.001
Some things I will do now that I previously felt were too risky.	70.7	29.3	74.0	26.0	56.8	43.2	18.71	<0.001
I prefer that my HIV-positive sex partners take anti-HIV medications so we don't have to bother with condoms.	86.4	13.6	91.1	8.9	67.5	32.5	86.99	<0.001
I prefer to use condoms even if my HIV-positive sex partner is taking anti-HIV medications.*	17.8	82.2	9.8	90.2	50.3	49.7	180.66	<0.001
I worry that anti-HIV medications do not completely eliminate the risk of getting HIV.*	11.6	88.4	7.9	92.1	26.7	73.3	87.3	<0.001
***Belief in Health Benefits of Treatments Scale***								
They are effective and they will extend HIV positive men's lives.	3.6	96.4	4.1	95.9	1.8	98.2	1.94	0.163
They improve HIV-positive people's health.	8.4	91.6	9.4	90.6	4.9	95.1	51.68	<0.001
They have serious side effects.*	29.6	70.4	25.0	75.0	47.2	52.8	30.86	<0.001
They are complicated to take.*	59.9	40.1	54.3	45.7	81.0	19.0	38.23	<0.001
They should be avoided until absolutely necessary.*	73.0	27.0	71.9	28.1	77.0	23.0	1.70	0.193
They are mostly easy to take.	31.0	69.0	35.9	64.1	12.9	87.1	68.0	<0.001
They have few serious side effects.	58.0	42.0	63.2	36.8	38.7	61.4	33.42	<0.001
They are toxic and will eventually damage people's health.*	54.6	45.4	53.3	46.7	59.6	40.4	2.06	0.151

* These items are presented in their original form in this table, but were reverse scored when entered into the relevant scale.

### Analysis

Survey data were analysed with Stata software (Version 12, Statacorp, Texas, USA). Items in our analyses included: age, education, ethnicity, HIV serostatus, social engagement with gay men, sexual identity, condom use with regular and casual partners, and various knowledge, belief and attitudinal items related to TasP, ART, and condoms as described above. For bivariate analyses, categorical variables were analysed using Pearson’s chi-square test or bivariate logistic regression, and t-tests were used for continuous variables. We used Type I error of 5% for these analyses. To identify independent associations with willingness to have serodiscordant CLAI if the positive partner was on ART, we used multivariate logistic regression and presented adjusted odds ratios (aOR) and 95% confidence intervals (95%CI). Associations with a p-value of less than 0.1 in bivariate analyses were block-entered into multivariate models.

## Results

Overall, 1,410 men accessed the survey, but 219 did not proceed beyond the first few screens, providing little or no information. Of the remaining 1,191 men, relatively complete data were received by 839 men on variables relevant to this analysis, including 165 HIV-positive men. Thus, a total of 571 men were excluded. Those who provided complete responses were more likely to be: older (mean = 39.5 vs 32.5, p<0.001), university educated (53.9% vs 38.3%, p<0.001), of Anglo-Celtic ethnic background (68.2% vs 42.4%, p<0.001), more socially engaged with other gay men (mean score 5.96 vs 5.28, p<0.001), and tested for HIV (91.8% vs 75.4%, p<0.001). All subsequent analyses were conducted on the restricted sample of 839 men.

The mean age was 39.5 years (median = 39 years). The vast majority of men identified as gay or homosexual (89.2%), with 9.0% identifying as bisexual, one man identifying as heterosexual, and 1.7% of men using other identities. Over two-thirds of the sample was of Anglo-Celtic ethnicity, and over half was university educated. About one-third (36.5%) reported that most or all friends were gay men, and 27.8% that they spent a lot of free time with other gay men. The majority of men reported being HIV-negative (70.1%), while one-fifth (19.7%) were HIV-positive, and 10.3% did not know their own serostatus. The large majority of HIV-positive men reported being on ART (84.2%) and reported having undetectable viral load (74.6%). Nearly half of the sample (44.7%) reported having a regular partner and 67.8% one or more casual partners in the previous six months. Of those with regular partners, 69.3% reported any CLAI-R; while 19.7% reported any CLAI with a serodiscordant or serononconcordant regular partner. Of those with casual partners, 46.9% reported any CLAI-C. One-quarter (25.3%) reported more than 10 sexual partners in the previous six months.

Men tended to disagree that TasP was sufficiently effective to justify reduced condom use (Table 1). HIV-positive men, however, were more convinced of TasP’s effectiveness. In general, men were far more convinced of the effectiveness of ART in treating HIV infection. When asked whether they would consider engaging in serodiscordant CLAI when the HIV-positive partner was on ART, only a minority (11.1%) of non-HIV-positive men agreed they would (Table 2). The majority of HIV-positive men (58.8%) agreed they would be willing. Respondents indicated they were more willing to consider serodiscordant CLAI if the HIV-negative partner was insertive and the HIV-positive partner receptive during anal sex, compared to the opposite scenario where the HIV-negative partner was receptive. Again, HIV-positive respondents indicated they were more willing to engage in CLAI in both scenarios compared to non-HIV-positive men.

**Table 2:** Treatment as prevention items and scales for total sample, and comparison between HIV-positive and non-HIV-positive men (bivariate).

	All men	Non-HIV-positive men	HIV-positive men	t or χ^2^	p-value
	n = 839	n = 674	n = 165		
Willingness to have serodiscordant condomless anal intercourse (CLAI) if the HIV-positive partner is taking ART–*n (%)*					
Any condomless anal intercourse (CLAI)	172(20.5)	75(11.1)	97(58.8)	184.74	<0.001
CLAI when HIV-negative partner is insertive	162(19.3)	73(10.8)	89(53.9)	160.01	<0.001
CLAI when HIV-negative partner is receptive	79(9.4)	30(4.5)	49(29.7)	106.89	<0.001
Knowledge of TasP research evidence–*n (%)*				48.14	<0.001
TasP has been proven effective in heterosexual sex only	68(8.1)	40(5.9)	28(17.0)		
TasP has been proven effective in homosexual sex only	12(1.4)	7(1.0)	5(3.0)		
TasP has been proven for any sexual situation, homosexual or heterosexual	211(25.2)	152(22.6)	59(35.8)		
TasP has not been proven effective for any population	178(21.2)	150(22.3)	28(17.0)		
Don’t know	370(44.1)	325(48.2)	45(27.3)		
Belief that HIV-positive men should go on ART to protect sexual partners–*n (%)*				18.67	<0.001
No	226(26.9)	200(29.7)	26(15.8)		
Yes, but only if ART is also good for their health	377(44.9)	284(42.1)	93(56.4)		
Yes, always	215(25.6)	170(25.2)	45(27.3)		
Not stated	21(2.5)	20(3.0)	1(0.6)		
Belief that HIV transmission is unlikely when an HIV-positive man is taking ART (i.e. TasP effectiveness)–*n (%)*				106.4	<0.001
Transmission is unlikely (TasP is effective)	183(21.8)	98(14.5)	85(51.5)		
Transmission is likely (TasP in not effective)	521(62.1)	459(68.1)	62(37.6)		
Don’t know	135(16.1)	117(17.4)	18(10.9)		
Belief in Effectiveness of TasP Scale–*mean (SD)*	1.83(0.54)	1.71(0.46)	2.30(0.58)	-13.90	<0.001
Belief in Health Benefits of Treatments Scale–*mean (SD)*	2.72(0.38)	2.66(0.35)	2.95(0.44)	-9.39	<0.001

Only a minority of men, and particularly the non-HIV-positive men, were aware of any evidence for TasP (Table 2). One-quarter incorrectly believed it had been proven effective in any sexual situation, homosexual or heterosexual, while one-fifth believed it had not been proven effective in any population. A small proportion correctly believed it had been proven only for heterosexual sex. Put another way, over one-quarter erroneously believed that TasP effectiveness evidence already existed for the gay male population at the time of the survey. HIV-positive respondents were more likely than non-HIV-positive respondents to believe TasP was proven in gay men, know that TasP had only been proven in heterosexuals at that time, and were less likely to state that they did not know about TasP.

One-quarter stated that HIV-positive men should not start ART to prevent HIV transmission to sexual partners, primarily because they believed treatments made no difference to the risk of passing on HIV (Table 2). While the majority of respondents said HIV-positive men should start ART for prevention, most of these agreed that they should only do so if starting ART was also good for the individual’s own health. HIV-positive respondents were more likely than other men to agree that HIV-positive men should go on ART for prevention, as long as ART was also good for the individual’s health.

Nearly two-thirds believed that TasP was not effective at reducing HIV transmission in gay men, while one-fifth believed it was effective and 16.1% stated that they did not know. HIV-positive men were more likely to believe that TasP was effective. The mean *Belief in Effectiveness of TasP Scale* score was 1.83 (SD = 0.19), indicating relatively sceptical beliefs about TasP. HIV-positive respondents had a higher mean score on the scale compared to non-HIV-positive men, and were more likely to have positive attitudes to TasP on each individual scale item (see Table 1).

The mean *Belief in Health Benefits of Treatments Scale* score for the whole sample was 2.72 (SD = 0.38), indicating relatively positive beliefs regarding the health benefits of ART. HIV-positive men had a higher mean score (Table 2). While the vast majority of respondents agreed that ART was effective and could extend the lives of HIV-positive people, there were also some mixed attitudes regarding treatment: the majority believed ART caused serious side effects, while relatively large minorities believed antiretroviral drugs were toxic, damaged people’s health in the long-term, were complicated to take, and that ART should be avoided until absolutely necessary (see Table 1). Compared to non-HIV-positive men, HIV-positive men generally held more optimistic attitudes on these items.

We compared men who stated they would be willing to have serodiscordant CLAI when the HIV-positive sexual partner was on ART to those who would not be willing (Table 3). In multivariate analysis, factors independently associated with willingness were: HIV-positive serostatus, reporting any serodiscordant or serononconcordant CLAI with a regular male partner in the previous six months, reporting any CLAI with a casual male partner in the previous six months, and having higher *Belief in Effectiveness of TasP Scale* scores. This analysis was also conducted using a similar outcome variable focusing on viral load, “willingness to have serodiscordant CLAI when the HIV-positive sexual partner had undetectable viral load” (data not shown). The results were statistically the same.

**Table 3:** Factors associated with willingness to have serodiscordant CLAI if the positive partner is taking ART (bivariate and multivariate).

	Unwilling	Willing	OR	95%CI	p-value	aOR	95%CI	p-value
	n = 667	n = 172						
***n (%)***								
HIV serostatus								
HIV-negative	522(78.3)	66(38.4)	Ref.	---	---	Ref.	---	---
HIV-positive	68(10.2)	97(56.4)	11.28	7.54–16.87	<0.001	3.65	2.06–6.46	**<0.001**
Unknown/untested serostatus	77(11.5)	9(5.2)	0.92	0.44–1.93	0.834	1.49	0.58–3.83	0.405
Belief: Research has proven TasP to be effective in gay men (no/yes)	145(21.7)	78(45.4)	2.99	2.10–4.25	<0.001	1.37	0.80–2.36	0.244
Belief: HIV-positive men should commence ART to protect sexual partners (no/yes)	446(66.9)	146(84.9)	2.78	1.78–4.35	<0.001	0.97	0.54–1.76	0.933
Any serodiscordant or serononconcordant CLAI with regular male partner (no/yes)	42(6.3)	32(18.6)	3.40	2.07–5.58	<0.001	2.18	1.08–4.38	**0.029**
Any CLAI with casual male partner/s (no/yes)	164(24.6)	103(59.9)	4.58	3.22–6.51	<0.001	2.10	1.30–3.42	**0.003**
Gay Sexual Identity (no/yes)	589(88.3)	156(90.7)	1.29	0.73–2.28	0.377	---	---	---
University Education (no/yes)	357(53.5)	95(55.2)	1.07	0.76–1.50	0.689	---	---	---
Anglo-Celtic Ethnicity (no/yes)	449(67.3)	123(71.5)	1.22	0.84–1.76	0.293	---	---	---
***mean (SD)***								
Belief in Effectiveness of TasP Scale	1.67(0.02)	2.44(0.37)	42.56	22.55–80.33	<0.001	21.39	10.78–42.44	**<0.001**
Belief in Health Benefits of Treatments Scale	2.67(0.01)	2.88(0.03)	4.24	2.60–6.93	<0.001	1.22	0.59–2.52	0.587
Gay Social Engagement Scale	5.84(0.06)	6.39(0.11)	1.25	1.13–1.40	<0.001	0.96	0.83–1.10	0.536
Age	38.4(0.52)	43.7(0.89)	1.03	1.02–1.04	<0.001	1.34	0.99–1.04	0.179

OR = odds ratio; aOR = adjusted odds ratio; 95%CI = 95% confidence interval; SD = standard deviation; CLAI = condomless anal intercourse; ART = antiretroviral therapy

## Discussion

Overall, most men stated that they would *not* be willing to engage in serodiscordant CLAI even if their HIV-positive partner was on ART, suggesting that most men tended not to trust TasP to protect themselves and their partners from HIV transmission. Similarly, the majority of men did not believe ART to be effective in reducing the risk of HIV transmission. As in other surveys that have examined aspects of gay men’s attitudes towards TasP in general, we found that most men, particularly most non-HIV-positive men, were sceptical about the overall effectiveness of TasP [10, 11, 16]. We explored this further by asking men directly about their views of acceptable sexual practices when HIV-positive partners are taking ART: What sexual practices would they find acceptable based on their own beliefs about TasP? Of course, an individual’s views of what they may or may not do in a future situation are hypothetical; nonetheless, an individual’s intentions regarding sexual behaviour do tend to predict actual behaviour [17], and provide a different perspective to examine attitudes. HIV-positive men indicated that they were more willing (56.4% vs 10.2%) to have CLAI with a serodiscordant partner in the context of TasP, as were those men who reported comparatively ‘riskier’ CLAI–either in the context of casual sex or with opposite-status or unknown-status regular partners. While more favourable views towards TasP were strongly related to willingness to have serodiscordant CLAI, attitudes towards ART for the purposes of treatment had no bearing upon willingness.

When considering their willingness to engage in CLAI based on knowledge of the HIV-positive partner taking ART, however, some GBM also seemed to take the relative riskiness of insertive and receptive sexual positioning into account [18]. So, for these men, it appeared that their willingness to engage in ART-protected CLAI was more likely if they also employed ‘strategic positioning’ as a method of risk reduction, although it should be noted that the survey did not explicitly ask about whether sexual positions were taken specifically to reduce the risk of transmission. In any case, they were more inclined to contemplate ART-protected CLAI when the negative partner was the insertive partner.

Men had a variety of accurate and inaccurate views about the research evidence for TasP, including over one-quarter who erroneously believed TasP’s effectiveness to already have been proven for sex between men in late 2012. Nearly half of the sample did not know of any TasP research at all, and a further one-fifth believed that TasP had not been proven effective in any population. HIV-positive men were both more likely to have correct knowledge of the research evidence (that is, that TasP had only been shown to be effective in heterosexual serodiscordant couples at this time) and to incorrectly believe it had been proven in gay men. Both of these may be explained by HIV-positive men having greater engagement with scientific findings compared to negative men. In particular, the Swiss Statement published in 2008 [19], while explicitly *not* about GBM, may have led to more optimistic beliefs about the status of the research evidence among some HIV-positive men. In any case, for many men, attitudes regarding TasP were formed in the context of mostly erroneous knowledge about the research evidence or a lack of awareness of the evidence. Of note, when controlling for other factors, belief that TasP had been proven effective in gay men was not associated with willingness to have serodiscordant CLAI when the HIV-positive partner was taking ART.

As has been found previously, most men were sceptical of the central concept that HIV treatments reduce transmission, although HIV-positive men tended to have more favourable attitudes towards TasP than HIV-negative and unknown-status men. Also similar to previous findings, most GBM supported the idea that HIV-positive men should start ART to reduce their infectivity, despite the general scepticism towards TasP [10, 11, 16, 20], but they only supported this if it benefitted the health of HIV-positive individuals. Recent findings indicating that early treatment is also beneficial for the individual HIV-positive person’s health [21] may provide a useful basis for health promotion work in this population, highlighting that ART has been shown to improve individual health and to reduce onward transmission at any stage of infection.

Given that it is a relatively new HIV prevention strategy, TasP may not be trusted partially because the suggestion that some forms of serodiscordant CLAI may be ‘safe’ contrasts heavily against community norms around the culture of condom use [11, 22] as well as seroadaptive strategies such as serosorting or negotiated safety, which by their very nature, aim to avoid serodiscordant CLAI entirely.

Taken together, these results present a complex picture of gay men’s TasP-related attitudes collected in 2012 when direct evidence for the effectiveness of TasP existed only for heterosexuals and prior to large-scale health promotion campaigns in the Australian gay community about TasP. Biomedical HIV prevention represents a rapidly changing environment with new research as well as community and policy responses emerging at a fast pace. For example, in 2014 and 2015, the first preliminary results regarding HIV treatment and transmission specifically within homosexual male serodiscordant couples were released [6, 7]. In Australia, there have also been major policy shifts towards TasP in several Australian states [23, 24] and the first explicit TasP-related community education campaigns promoted to gay men (see www.endinghiv.org.au).

While it is not yet clear what impact these developments have had on gay men’s attitudes, especially recently, Holt and colleagues [11] have argued that TasP did not appear to be an acceptable strategy to most gay men, although HIV-positive men, those with HIV-positive partners, and men who had engaged in relatively risky behaviour had more positive views. Highlighting the personal health benefits of treatment for HIV-positive people may provide a good basis for discussing the benefits of TasP, which is currently being implemented in a landscape of very uneven knowledge, attitudes, social norms, and behaviours in the gay community. For most GBM, lack of acceptance of TasP might be due to an ongoing commitment to condom use. For these men, TasP need have no effect on their behaviour, but nonetheless, the potential positive implications of TasP do need to be more widely understood. Data from clinical research studies indicate that in the context of limited knowledge and widespread scepticism, relatively few men contemplate incorporating TasP into their considerations. Promisingly though, those that are engaging in practices that have an elevated risk of transmission tend to be more inclined to incorporate TasP into their thinking. This implies that they need support to better understand the conditions under which TasP is most effective (for example, sustained undetectable viral load and not having any sexually transmitted infections). It also suggests that HIV-positive men in particular would most benefit from reassurance.

In both research and health promotion, there has been little focus on those men who do appear to find TasP an acceptable strategy. More specific information is needed on what these men are doing and in what contexts. Research is increasingly supporting the efficacy of TasP in the specific context of male serodiscordant relationships, where the limited available evidence to-date suggests that CLAI with a seropositive partner on ART and with undetectable viral load presents a very low risk for transmission [6, 7]. Although at the population level there may be a link between increased treatment coverage and decreased HIV incidence over time, individual gay men need to consider many complex issues when trying to incorporate TasP into their existing repertoire of transmission-reduction strategies. In the context of ongoing relationships, as with ‘negotiated safety’ [25], the HIV-negative partner would presumably have a greater level of knowledge about his HIV-positive partner’s testing patterns and results than would be the case for casual sex partners. For these reasons, again much like negotiated safety, TasP may have the most promising individual-level utility for men in serodiscordant couples where the HIV-negative partner is *not* also engaging in CLAI with outside casual partners. For HIV-negative men who do engage in CLAI with casual partners, PrEP is likely to be the most protective strategy rather than relying on ‘viral load sorting’ with casual partners [26]. Emerging research suggests that use of such biomedical strategies may be becoming increasingly common among gay men, and has identified a new strategy termed ‘biomed-matching’ (where individuals have CLAI only when both individuals are on PrEP or have undetectable viral load) [27]. In any case, the minority of men already, or considering, engaging in TasP-related risk reduction practices have not been provided with any clear guidance on how to make ‘viral load sorting’ or ‘biomed-matching’ safer if they choose to engage in it.

Participants in this community-based study were broadly similar to other samples of Australian gay men [28, 29], albeit with a somewhat higher proportion of HIV-positive men. Nonetheless, it was a volunteer, online convenience sample and is unlikely to be representative of all homosexually-active men in Australia [30]. Online samples of GBM may over-represent GBM who are more sexually active as they commonly use online dating sites to meet sex partners [28]. This analysis excluded over five hundred men who did not provide complete responses. This may have been because respondents were being asked about their knowledge of and attitudes toward technologies and interventions that had not yet been made available, at least in Australia, and about which there had as yet been little community discussion. Participants who were older, more educated, of Anglo-Celtic background, more socially engaged with other gay men, and tested for HIV were more likely to have provided complete responses; these men may have had different, perhaps higher, levels of knowledge. The generalisability of the findings must be considered with regard to this limitation. Furthermore, the generalisability of these findings to other contexts may be limited by differences between Australia and other locations: Testing for HIV among Australian GBM and uptake of ART among HIV-positive men is relatively high compared with some similar countries [31, 32], and the use of viral load testing for HIV clinical monitoring is standard-of-care [33]. As a cross-sectional survey it is not possible to determine any causal relationships in the data. For example, men may make decisions about behaviour on the basis of attitudes towards issues such as TasP, or it may be that men who want to engage in CLAI develop attitudes to support that behaviour. The low level of detailed knowledge and incorrect assumptions about the research evidence could be taken to support this view. Additionally, non-HIV-positive men’s willingness to state that they would engage in ART-protected CLAI with an HIV-positive partner may have been impacted by their willingness to have sex with an HIV-positive partner at all; such stigma is well-documented [34–36].

In summary, our findings indicated that most GBM in Australia were sceptical of TasP, although HIV-positive men and men who engaged in riskier sexual behaviour were less sceptical. Knowledge of research evidence was inconsistent. Greater willingness to engage in CLAI with a serodiscordant partner was associated with more favourable attitudes towards TasP, particularly among HIV-positive men and those men who had already engaged in some serodiscordant or nonconcordant CLAI. Community education campaigns explicitly discussing TasP have recently been implemented in some parts of Australia, providing general information about the research evidence. However, for GBM with serodiscordant sexual partners to successfully apply TasP to reducing transmission risk, more support and education is needed to enable better utilisation of TasP in specific relational and sexual contexts.
